# Sea lice infestation dataset for wild and farmed salmon populations on the Pacific coast of Canada (2001–2023)

**DOI:** 10.1038/s41597-025-05653-x

**Published:** 2025-07-31

**Authors:** Crawford W. Revie, Thitiwan Patanasatienkul, Gregor McEwan, Martin Krkošek, Lance Stewardson

**Affiliations:** 1 Department of Computer and Information Sciences, University of Strathclyde, 26 Richmond Street, Glasgow, G1 1XQ Scotland UK; 2 Department of Health Management, University of Prince Edward Island, 550 University Avenue, Charlottetown, Prince Edward Island C1A 4P3 Canada; 3 WOAH Regional Representation for Asia and the Pacific, Bunkyo-Ku, Tokyo Japan; 4 Modail Mara, Charlottetown, PEI Canada; 5 Department of Ecology and Evolutionary Biology, University of Toronto, 1310 Marwalk Cres, Toronto, ON M5S 3B2 Canada; 6 Mainstream Biological Consulting, 1310 Marwalk Cres, Campbell River, BC Canada

**Keywords:** Evolutionary ecology, Marine biology

## Abstract

Monitoring sea lice infestation levels on populations of farmed and wild salmonids is critical to the development of evidence-based policy designed to mitigate the risk these ectoparasites represent to wild juvenile salmon and the on-going sustainability of salmon aquaculture. The data described relate to sea lice monitoring along the coast of British Columbia (BC), Canada from all areas where Atlantic salmon farms are present, spanning over two decades of observations from these farms and adjacent wild Pacific salmonid populations. Around 10,000 mean monthly sea lice estimates are included from almost 100 salmon farms spread across seven ‘fish health’ zones along the BC coast. Sea lice infestation data from over 365,000 wild hosts, observed as part of almost 17,000 sampling events in these zones, are also reported. While observations were made in the same broad geographical area, temporal coverage varies by zone. These data provide valuable insights into long-term trends, including spatial variability and demographic patterns within the sea lice populations observed on various host species along the BC coast.

## Background & Summary

Infestation of salmonids by marine ectoparasitic copepods, commonly referred to as “sea lice”, remains a significant challenge to sustainable Atlantic salmon aquaculture^1^. In the case of farmed hosts, significant levels of infestation can lead to reduced growth, poor feed conversion efficiency, increased stress and in extreme cases, mortality^2^. In locations where significant stocks of wild salmonids are present, a clear understanding of sea lice infestation patterns in both wild and farmed salmon populations is of paramount importance due to potential spill-over effects, which has been identified as a conservation issue in a number of countries^3–8^.

These challenges have been identified over the past two decades in British Columbia and a wide range of studies have explored both the patterns of infestation^9,10^, the potential linkages between farmed and wild populations^11–14^, and the likely effects of these interactions^15,16^. It is not the purpose of this paper to engage with this broader set of debates, other than to note that in a number of these studies general claims are made about the situation for the whole of the BC coast based on rather limited datasets, either in terms of spatial coverage or temporal range. Papers that cover a wider spatial and/or temporal range, illustrate the range of variation that is typically found when considering infestation patterns at these larger scales^9,17^.

In some cases, data reported within these studies have been made available, or make reference to open access sites (e.g. from government reports^18^ or from NGO groups^19^). However, due to the variety of groups involved in sea lice monitoring, as well as changes in practice over time even within the same group, there tend to be differences in the formats of these data. In the case of farmed data, for example, the sea lice data on the official Fisheries and Oceans Canada (DFO) site noted above consisted of monthly mean values when reporting started in 2010, but switched to weekly means in 2013; over time there was also a shift from farm-level averages to pen-level data. Similar differences can be observed over time in the data reported on the websites maintained by the various aquaculture operators in BC. In the case of data reported from observations on wild Pacific salmonids the differences are even more marked. For example, in the early data reported here, limited details exist relating to sea lice species, particularly for parasites at an early stage of development. Where fish are observed in the field, as opposed to those which were lethally sampled and sent to a laboratory for identification of sea lice infestation, there are inevitable limits to the level of detail that it is possible to provide in terms of the sea louse species and/or stages that may be present on a fish.

One of the key objectives when creating this dataset was to find data formats that allowed for maximal temporal and spatial comparison while maintaining as much detail as was possible from the original datasets. It is our view that the debate around salmon farming policy in British Columbia has sometimes used small-scale or anecdotal findings to make overly generalised statements about the sector. The long-term dataset reported here can be used to support a clearer and more comprehensive understanding of sea lice infestation patterns along the BC coast over the past two decades. In addition, recent government policy has already led to a significant reduction in the number of salmon farms in at least two areas covered by this dataset. Exploring the impact of such policy decisions represents another important use case for these data.

## Methods

### Geographic extent

**Fig. 1:**
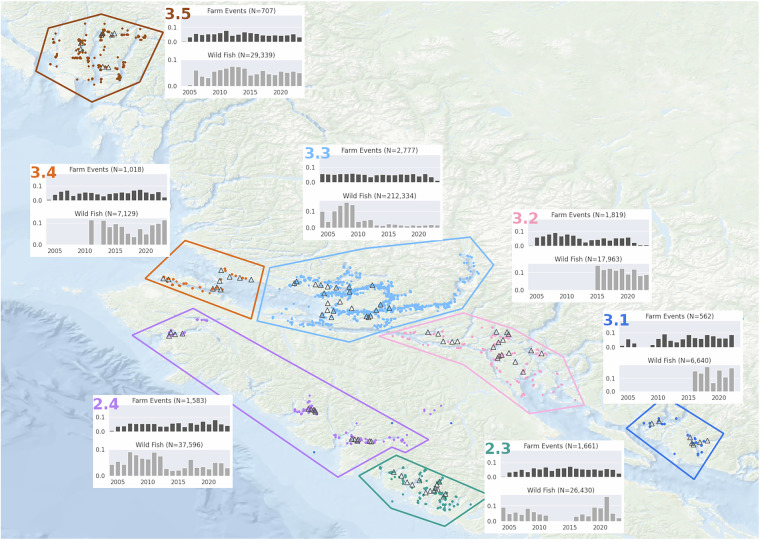
Map of the BC coastal area showing the location of farms sites (open triangles) and wild sampling sites (closed circles) from which data were obtained over the period 2001 to 2023. The panels for each fish health sub-zone illustrate the proportion of data from that zone obtained in each year from 2004 onwards from both farmed and wild sources. (The numbers of farm sampling events and wild fish observed are shown in the upper and lower panels for each sub-zone).

The data come from locations along the coast of BC, Canada. The responsible regulatory body, Fisheries and Oceans Canada (DFO), has historically used nine ‘fish health’ sub-zones to divide up this coastal region^20^, based on two larger salmonid fish health zones (Zone 2: Vancouver Island, and Zone 3: Mainland Coast). Within Zone 2 only two sub-zones (2.3 and 2.4) have active Atlantic salmon fish farms, while in Zone 3 all five sub-zones (3.1 to 3.5) have active farms. These seven sub-zones are shown in Fig. 1 and present a summary of the locations from which the data in this dataset were collected. The open triangles represent the location of Atlantic salmon farms from which data have been included, while a solid circle represents each location at which observations of sea lice on wild Pacific juveniles were made. Information panels are provided for each sub-zone, indicating the total number of records and temporal extent of data from farm and wild sea lice monitoring activities that have been included in this dataset. As can be seen from these summary panels, the temporal coverage of data from salmon farms has remained relatively consistent over the past two decades, while sampling from wild populations has varied over space and time. Those familiar with the most recent changes in the aquaculture sector in BC will not be surprised to see declines since 2021/2022 in the number of “farm events” (sea louse sampling events) being reported from the Discovery Islands (3.2), as well as the Broughton Archipelago (3.3), as a consequence of the reduced number of Atlantic salmon farms operating in those areas.

### Data sampled on wild populations

The dataset relating to wild observations is based on samples taken during the out-migration of wild juvenile fish from rivers to the Pacific Ocean stretching from March to July each year, but with the majority of observations (well over 90%) taking place in April, May and June (see Table 6). As can be seen from Table 1a, by far the most commonly observed wild Pacific species were juvenile chum (*Oncorhynchus kata*) and pink (*O. gorbuscha*) salmon, which between them account for around 93% of all fish sampled. Three-spined sticklebacks (*Gasterosteus aculeatus*) account for a further 3% of the observations, while each of the other Pacific salmon species typically represent little more than 1% of the samples (with the exception of 2003, when an anomalously high proportion of coho, chinook and “other” species were present in the dataset, due to a different sampling protocol followed by DFO in that year).

**Table 1:** **a** Total number of sampling events and wild fish assessed for sea lice infestation on the BC coast by host species in each of the sampling years. **b** Total number of wild fish assessed for sea lice infestation on the BC coast as represented within the various programmes active over the range of sampling years.

Year	All events	Events with fish	Chum	Pink	Coho	Chinook	Sockeye	Stickleback	Pacific Herring	Other species	All fish
2001	16	16	—	268	—	—	—	—	—	—	**268**
2002	31	31	67	497	—	—	—	—	—	—	**564**
2003	1,807	1,273	15,484	10,220	2,909	1,011	64	2,886	828	1,614	**35,016**
2004	1,109	799	19,698	3,253	104	754	2	1,479	93	1	**25,384**
2005	969	599	4,955	6,091	128	405	271	45	—	1	**11,896**
2006	1,098	707	14,267	11,355	170	214	9	10	—	695	**26,720**
2007	1,127	943	18,770	13,907	165	345	50	1,350	—	1	**34,588**
2008	1,214	953	15,785	20,920	190	521	10	1,601	—	—	**39,027**
2009	1,168	960	15,699	16,680	136	126	2	2,567	6	65	**35,281**
2010	732	603	5,991	7,381	226	137	84	26	—	—	**13,845**
2011	852	669	8,062	8,017	320	181	1	55	60	18	**16,714**
2012	543	491	7,841	6,961	330	137	206	27	—	—	**15,502**
2013	225	215	3,981	3,694	66	18	26	—	—	7	**7,792**
2014	191	179	2,908	3,644	58	27	55	—	1	1	**6,694**
2015	460	331	3,542	3,892	370	129	4,113	24	419	1	**12,490**
2016	535	372	5,604	4,022	377	230	2,635	8	159	25	**13,060**
2017	552	476	8,136	3,316	374	45	881	11	58	1	**12,822**
2018	556	395	5,025	3,864	200	99	444	41	52	3	**9,728**
2019	774	448	6,797	3,315	370	350	769	104	128	—	**11,833**
2020	700	468	5,873	4,364	332	275	264	4	10	1	**11,123**
2021	828	595	8,668	5,471	180	279	157	189	22	5	**14,971**
2022	752	512	7,093	4,772	302	171	43	—	1	1	**12,383**
2023	681	430	3,639	4,884	238	99	198	—	3	2	**9,063**
**Total**	**16,920**	**12,465**	**187,885**	**150,788**	**7,545**	**5,553**	**10,284**	**10,427**	**1,840**	**2,442**	**376,764**

Year	SCS	MK	Hak	CC	Kit	DFO	BAMP	MERP	Pacif	MBC	All fish
2001	268	—	—	—	—	—	—	—	—	—	**268**
2002	564	—	—	—	—	—	—	—	—	—	**564**
2003	676	4,333	—	—	—	29,472	—	535	—	—	**35,016**
2004	1,087	11,575	—	—	—	8,657	—	4,065	—	—	**25,384**
2005	2,084	—	—	—	192	6,198	—	3,422	—	—	**11,896**
2006	1,708	12,609	—	—	1,816	7,360	—	3,227	—	—	**26,720**
2007	1,650	17,829	—	—	1,132	9,378	—	4,599	—	—	**34,588**
2008	2,345	21,361	—	—	954	9,170	—	5,197	—	—	**39,027**
2009	2,549	16,577	—	—	1,675	10,320	—	4,160	—	—	**35,281**
2010	2,276	—	—	—	1,852	—	6,090	3,627	—	—	**13,845**
2011	3,300	—	—	—	2,031	—	6,164	5,219	—	—	**16,714**
2012	2,979	—	—	—	2,203	—	7,467	2,853	—	—	**15,502**
2013	3,780	—	—	—	2,190	—	—	1,822	—	—	**7,792**
2014	3,356	—	—	—	1,989	—	—	1,349	—	—	**6,694**
2015	2,771	—	6,794	—	1,230	—	—	1,695	—	—	**12,490**
2016	2,698	—	4,435	—	1,355	—	—	598	—	3,974	**13,060**
2017	2,170	—	1,595	—	1,753	—	—	—	378	6,926	**12,822**
2018	1,578	—	1,205	172	1,353	—	—	—	260	5,160	**9,728**
2019	2,334	—	1,628	1,553	1,277	—	—	—	520	4,521	**11,833**
2020	1,989	—	444	993	1,652	—	—	—	355	5,690	**11,123**
2021	2,367	—	375	2,235	1,543	—	—	—	642	7,809	**14,971**
2022	2,944	—	285	—	1,657	—	—	—	696	6,801	**12,383**
2023	2,457	—	484	—	1,485	—	—	—	—	4,637	**9,063**
**Total**	**49,930**	**84,284**	**17,245**	**4,953**	**29,339**	**80,555**	**19,721**	**42,368**	**2,851**	**45,518**	**376,764**

It can also be seen from Table 1a that the number of fish sampled varied over the years, ranging from over 6,500 to almost 40,000; the exception being 2001 and 2002 where many fewer fish (primarily pink salmon) were observed, from just one of the sampling programmes. In general, these variations are due to the fact that different monitoring programmes existed over this time period. More information relating to each of the programmes listed in Table 1b is provided in the notes that accompany the data set, but these broadly fell into one of two main categories: ‘non-lethal’, where the fish are observed *in-situ* at the sampling site, and ‘lethal’, where the fish are retained at the sampling site and sent to a laboratory for assessment. Details on the specific protocols can be found both for non-lethal sampling^16,21^ and for lethal sampling^9,15,22^. The sampling programme with the longest temporal extent is that carried out by the Salmon Coastal Station (“SCS” in Table 1b), though this is limited to three sites in a single zone (3.3: Broughton Archipelago). The research programme co-ordinated by Dr. Martin Krkošek (“MK” in Table 1b) between 2003 and 2009, contributed a significant number of samples but was again limited to the Broughton. A more limited number of wild fish were sampled by the Cedar Coast Field Station (“CC” in Table 1b) in the Clayoquot Sound (sub-zone 2.3) between 2018 and 2021. All of these programmes largely adopted a similar ‘non-lethal’ protocol. Most of the remaining programmes adopted the ‘lethal’ protocol, with sea lice identification taking place in a laboratory, and were sponsored by local first nations (“Kit”), the Canadian government (“DFO”), the BC aquaculture sector (“MERP”, “MBC”, “Pacif”), or through co-operation among these groups (“BAMP”). The final programme integrated into this coast-wide dataset was run by the Hakai Institute (“Hak”) and adopted differing protocols (both ‘non-lethal’ and ‘lethal’) in different studies over time. Details relating to each of these programmes / protocols can be found in Table S1.

**Fig. 2:**
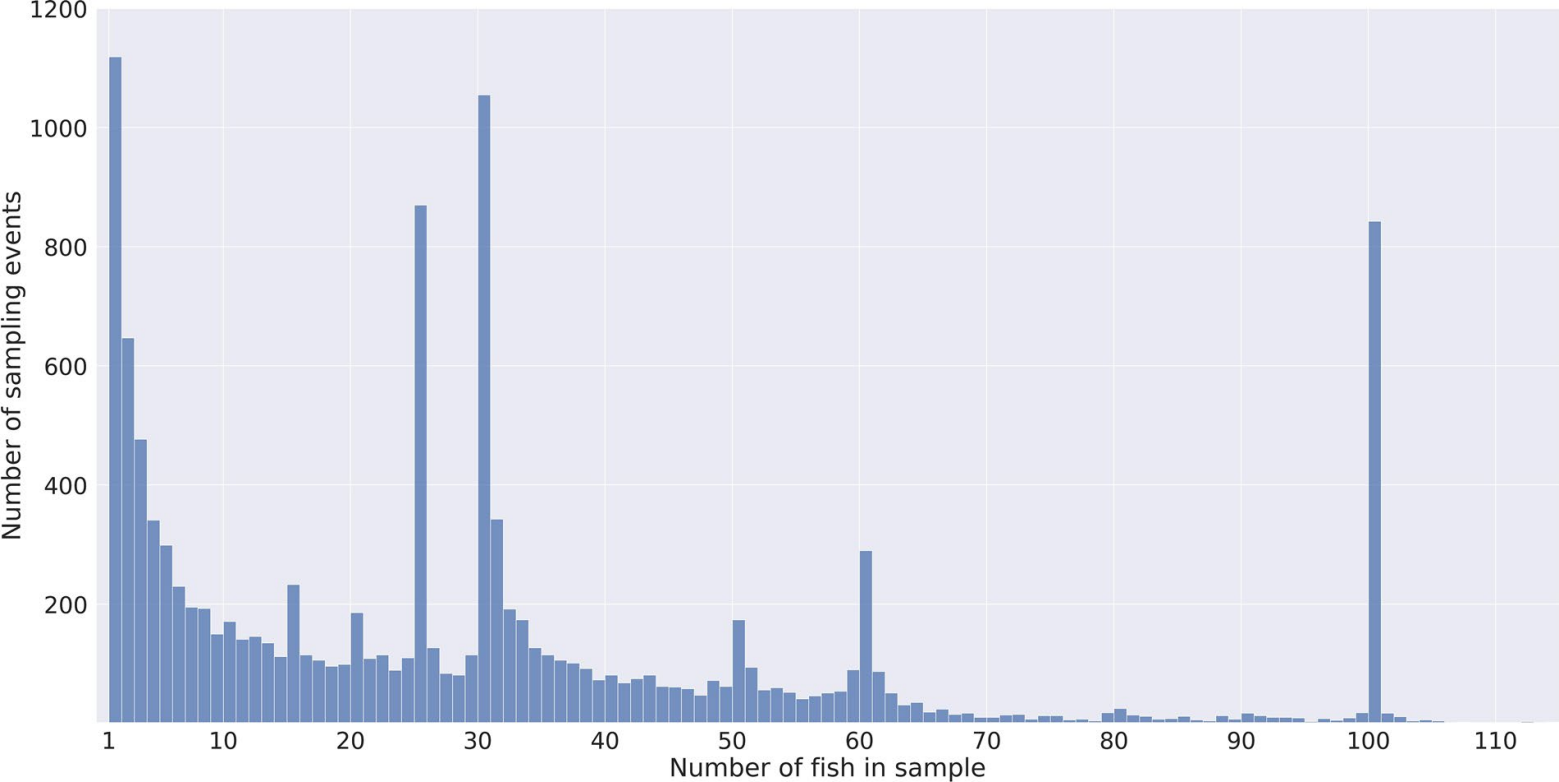
Frequency plot indicating the number of fish assessed in the 12,465 sampling events where fish were caught. (In almost 4,500 sampling events no fish were caught/assessed. There were also around 15 events where more than 115 fish were included in the sample – see code and output in *Fig2_fish_per_sample.ipynb* for details).

Field sampling took place weekly, biweekly, or monthly, depending on the programme, with the specific observation date and geographical coordinates of every sampling event being recorded. Each fish observed was measured for fork length (mm) and, in the case of the majority of those that used a ‘lethal’ protocol, weight (g) was also recorded. The numbers of fish observed during each sampling event varied according to location and timing within the season. Indeed, as can be deduced from Table 1a, that just over 25% of all attempts to sample wild juveniles resulted in no suitable fish (i.e. those in the target groups of Pacific salmonids, three-spined sticklebacks or herring) being caught. It can also be seen from the distribution plot shown in Fig. 2 that a fair proportion of sampling events resulted in only a few fish being assessed. In terms of the maximum number of fish assessed, under the non-lethal collection protocols, up to around 100 fish were assessed, as can once again be seen in Fig. 2. In early studies using the lethal protocol up to 30 fish from the most commonly occurring species (chum or pink) were assessed, though in many cases only one of these two species was present, which accounts for the peaks seen at 30 and 60 fish in Fig. 2. In later studies using lethal sampling, to avoid unnecessary death of fish, the numbers of a single species that were retained in some regions was reduced to 25 and then to 15, and once again the associated ‘peaks’ associated with these values can be seen in Fig. 2. In total well over 1 million wild fish were sampled across these wild sampling programmes, but the maximum levels put in place for active assessment noted above meant that only roughly 25% of these fish were assessed for sea louse infestation.

The differing field protocols also led to differences in how sea lice infestation data were recorded. In the case of laboratory-based assessment (under the ‘lethal’ protocol), typically the developmental stage (copepodite, chalimus, pre-adult, or adult), species (*Caligus clemensi*, *Lepeophtheirus salmonis*, or not identified to species), as well as the sex (for motile stages) of any sea louse that was observed were recorded. However, those who are aware of the recent history of the *L. salmonis* species will know that only two chalimus stages are now recognised^23^, so even where stage data had been noted, records prior to 2014 which indicated “Chalimus 1” or “Chalimus 2” were updated to “Chalimus 1”, and those that had been “Chalimus 3” or “Chalimus 4” to “Chalimus 2”. In the case of the ‘non-lethal’ protocol, sea lice were categorised to broad development stage, but typically the chalimus lice stages were unidentified to species. Irrespective of the specific protocols adopted, we have attempted to maintain as much detail as was available, with the inclusion of codes to allow for categorisation into broader ‘common groupings’ where comparisons were being made across multiple protocols.

### Data collected from salmon farms

The second major component of this dataset relates to information obtained as part of routine sea lice monitoring from just under 100 Atlantic salmon farms along the BC coast that have been in operation at various points over the past two decades. Not all of these farms are currently operating and, for some sites, data only exist for a few years; summary details based on each DFO fish health sub-zone can be seen in the information panels of Fig. 1. At the farm level, the main data recorded are the sea lice infestation levels at a given time on that farm. A number of pens, typically 2 to 3, are sampled each week and sea lice infestation data are recorded at the pen level. Typically, 20 fish are taken from each pen, resulting in estimates based on between 40 to 60 fish each week, or around 160 to 240 fish each month. At each pen-level event, fish are extracted into a tote where anaesthesia is typically applied to facilitate handling and examination without causing undue stress. In the dataset provided here, a mean monthly abundance value was calculated, based on the total sea lice counts divided by the number of sampled fish, for each month that a farm was in operation. Counts by developmental stage and sea lice species were recorded. All copepodite and chalimus are grouped as “chalimus”. Only motile sea lice were identified to species (*C. clemensi* or *L. salmonis*), while sex was also recorded for motile *L. salmonis*.

The size of each farm (in terms of farmed fish inventory) relative to all those operating within a given zone for a given month is captured, which allows a properly weighted average to be estimated from all farms within a zone. Even this does not allow for analyses that wish to come up with some estimate of the total numbers of sea lice from a particular farm. However, commercial sensitivity and legal restrictions mean that detailed inventories cannot be disclosed at the individual farm level. What has been done instead is that an average ‘load’ value is reported for each zone/month. This is the median ‘load’ value of estimated total sea lice at a specific stage from all sites in a given zone; where these totals are in turn based on the mean monthly abundance multiplied by the (known, but un-reported in this dataset) estimated farmed salmon inventory on that site in a given month.

The figures shown in Table 2 illustrate how these data could be used to review sea lice trends seen on salmon farms over the two decades under consideration. For each zone, an annual value is shown which represents the weighted mean *L. salmonis* motile infestation level based on data from all farms in that zone over the period from March to June (the ‘sensitive period’ defined within DFO regulations, to coincide with the main period of wild juvenile salmon outmigration). The number of farms from which data have been taken and the total number of monthly counts used in these estimations are also shown at the foot of the table.

**Table 2:** Weighted mean *L. salmonis* motile infestation abundance levels across all salmon farms by fish health zone, based on the wild juvenile salmon outmigration period (March–June) each year. (Entries that were unusually elevated are indicated with an *).

Year	DFO Health Sub-Zone						
	2.3	2.4	3.1	3.2	3.3	3.4	3.5
2004					4.53*		
2005	1.02	0.48	0.11	1.58	1.37*	0.72	0.23
2006	2.14	0.60	0.12	1.46	0.90	0.88	0.16
2007	1.05	0.50		0.53	0.46	0.97	0.20
2008	1.04	0.40		1.72	0.27	0.90	0.24
2009	0.58	0.58	0.20	0.67	0.15	0.97	0.43
2010	0.36	0.48	0.21	0.62	0.43	1.25	0.60
2011	0.42	0.79	0.12	0.85	0.18	0.32	0.30
2012	0.39	0.64	0.08	1.27	0.34	0.72	1.19
2013	0.70	1.29	0.02	0.30	0.29	0.58	4.94*
2014	0.74	0.39	0.08	0.85	0.50	0.16	0.96
2015	1.27	3.18*	0.08	2.56*	1.19*	1.71*	7.06*
2016	1.14	2.58	1.19	1.06	0.51	0.97	0.77
2017	1.35	0.66	0.42	0.58	0.25	0.69	1.10
2018	7.73*	1.28	0.14	0.20	0.28	0.48	0.51
2019	1.21	0.52	0.29	1.04	0.46	0.65	0.51
2020	1.40	2.94*	0.93	1.62	0.17	0.81	1.52
2021	0.72	0.28	0.04	1.31	0.66	1.60*	1.19
2022	2.13	1.06	0.99	1.23	0.59	0.91	1.86
2023	0.16	0.26		1.64	1.06	0.76	0.82
**Median**	**1.04**	**0.60**	**0.13**	**1.06**	**0.46**	**0.81**	**0.77**
Monthly counts (N)	609	604	224	605	979	347	259
Farms (N)	14	15	7	19	23	10	8

In classical statistics the definition of an ‘outlier’ is any point lying more that 3 standard deviations from the mean, and such points are often removed from further analyses. We do not believe that excluding such values can be justified, but it can be useful in identifying ‘unusual’ data points such as the four cells (shown with an * and in **bold**). In addition, a slightly broader definition of ‘unusual’ could be taken to include any point lying more than 1.5 standard deviations away from the mean; the cells which met this criterion are also shown with an *. It seems clear that something unusual in terms of sea lice infestation on farms appears to have occurred in 2015, with all zones other than 2.3 and 3.1 (the most southerly zones) exhibiting unusually high levels of infestation. This was noted in a more fine-grained analysis, based on DFO published data over the period 2011 to 2016^24^, and in that analysis was largely attributed to the so-called ‘warm blob’^25^, that it is estimated had its most significant impact in the ocean around Vancouver Island in 2015. It is interesting to note that there appear to be no other years over these two decades in which a similar widespread increase was observed, though given the ocean-wide impact that the ‘blob’ is thought to have exerted, it is not obvious why two zones would appear not to have been similarly affected in 2015.

In addition, a set of data curated by DFO from sea lice counts provided to them by aquaculture operators, as the regulator in BC, has also been included. These data are typically recorded and submitted to DFO at weekly intervals and when aggregated over year by zone, will give estimates similar to those shown in Table 2, though without the relative weighting for differing numbers of fish on the various sites involved in estimating each mean value. (See Table S2 for details.)

## Data Records

The dataset is available at 10.6084/m9.figshare.28078100^26^, with this section being the primary source of information on the availability and content of the being described. There are five files associated with these data, two relating to sea lice observations on wild fish and three relating to sampling for sea lice abundance on Atlantic salmon farms. Table 3a describes the data fields associated with each wild sampling event, which includes the date and location of each observation and the observation programme (“Source”) under which each event was carried out. The file consists of 16,920 rows and 10 columns, where each row represents a field sampling event. Table 3b describes the data fields associated with the sea lice observations made on each wild fish. In addition to the 12 fields that are used to record sea lice presence according to various species and stages of life cycle development, the host species and physical characteristics are noted, as well as a reference field (“Event_ID”) to link each wild fish record to the field event during which it was sampled. This file contains a total of 376,764 rows, with each row representing a single fish.

**Table 3:** **a** Description of data fields associated with each wild sampling event [***all_wild_sample_events***]. **b** Description of data fields associated with each wild fish that was assessed for sea louse infestation [***all_wild_fish_lice***].

Field	Description
event_id	A unique ID associated with each wild sampling event
sampledate	Date when the sampling event took place (dd/mm/yyyy)
dfozone	One of seven fish health zone codes where the event took place. There were also a group of events collected by the Hakai Institute at various points along the Johnstone Straight. These do not fit neatly into a single existing sub-zone and so this field has been left blank for those events, while their ‘region’ (see next field) attribute has been noted as “BA/DI border”.
region	One of fourteen areas along the BC coast. (Some DFO zones have only one region, e.g. {3.4} = {Port Hardy}, while others have several, e.g. {2.4} contains {Esperanza} {Espinosa} {Muchalet} {Quatsino}.)
sample_site	A textual name that was used to describe the specific sampling site
latitude	Latitude of sampling location in decimal degrees (to 4 decimal places)
longitude	Longitude of sampling location in decimal degrees (to 4 decimal places)
source	One of ten textual values representing the programme or organisation responsible for the sampling event. (See Table S1 for details relating to each of these programmes and their protocols.)
source_code	A short-hand label by which to refer to the ‘sources’ noted above.
protocol	One of three labels {Lethal} {Non-lethal} {Mixed} used to indicate the host capture and sea lice enumeration method adopted for a particular event. (See Table S1 for further detail.)

Field	Description
event_id	ID that associates each fish with the event in which it was sampled
fish_id	A unique ID associated with each wild fish that was sampled
length	The measured fork length for each fish (in mm)
weight	The weight for each fish (in g)
height	The height (vertical measurement from the bottom of the belly to the top of the back) for each fish (in mm)
fish_species	One of eight fish species designation to which each fish was allocated
lep_cop	Number of sea lice identified as L. salmonis species at copepodid stage
lep_chal	Number of sea lice identified as L. salmonis species at chalimus stage
lep_motile	Number of sea lice identified as L. salmonis species at motile stage
lep_unknown	Number of sea lice identified as L. salmonis species but unidentified stage
cal_cop	Number of sea lice identified as Caligus species at copepodid stage
cal_chal	Number of sea lice identified as Caligus species at chalimus stage
cal_motile	Number of sea lice identified as Caligus species at motile stage
cal_unknown	Number of sea lice identified as Caligus species but unidentified stage
unknown_cop	Number of sea lice at copepodid stage but not identified to species
unknown_chal	Number of sea lice at chalimus stage but not identified to species
unknown_motile	Number of sea lice at motile stage but not identified to species
unknown_unknown	Number of sea lice at an unidentified stage and not identified to species

Table 4a describes the data fields associated with each Atlantic salmon farm from which sea lice observations have been included. This includes the farm’s name, location and the aquaculture company responsible for that site. The file consists of 96 rows and 7 columns, with each row representing a different farm. Table 4b describes the data fields associated with the monthly mean abundance values for sea lice levels observed on farmed fish. The relevant farmed site, sampling year and month are noted, together with four columns providing mean sea louse abundance values and the number of sampled fish from which these means were generated, as well as a field indicating the ‘weighting’ that should be given to this monthly value when estimating zonal averages, based on the proportional number of fish present on that farm compared to the whole zone. There are 10,159 rows, each one representing a single monthly farm record. Table 4c describes a set of data fields that are derived from the farm-based sea lice abundance values and attempt to give a sense of the overall zonal sea louse ‘load’. In this case, each of the 1,527 rows contains information on the zone, sampling year and month, with the four sea lice columns representing the median total load of each species/stage recorded, where each farm’s load is estimated based on the monthly mean abundance on that farm multiplied by the estimated number of fish present on that farm during the month under consideration.

**Table 4:** **a** Description of data fields associated with each Atlantic salmon farm operating on the BC coast [***industry_farm_details***]. **b** Description of data fields associated with the mean monthly sea lice abundance estimates reported from each farm [***industry_farm_abundance***]. (An additional file covering similar data, but typically at a weekly level and published by DFO since 2011, has also been included. See Table S2). **c** Description of data fields associated with the estimated median monthly sea lice ‘load’ associated with each DFO fish health zone [***industry_zone_loads_median***].

Field	Description
facility_id	A unique ID associated with each farm
name	The textual name used to refer to each farm
dfozone	One of seven fish health zone codes where the farm is located
latitude	Latitude of farm location in decimal degrees (to 4 decimals places)
longitude	Longitude of farm location in decimal degrees (to 4 decimal places)
company	The textual name of the owner/operator of a given farm
region	One of ten areas along the BC coast

Field	Description
facility_id	ID that associates each record with the farm from which the data comes
year	The year to which the abundance data relate (yyyy)
month	The month for which the abundance data have been estimated (mm)
fish_selected	The number of fish involved in estimating the monthly abundances
chalimus_ab	Mean monthly abundance of all sea lice in a stage prior to becoming motiles
lep_motile_ab	Mean monthly abundance of L. salmonis in all motile stages (including L. salmonis in the adult female stage)
lep_af_ab	Mean monthly abundance of L. salmonis in the adult female stage
cal_motile_ab	Mean monthly abundance of Caligus sea lice in the motile stage
weight	A four-digit decimal, giving the proportional weighting of this farm within the zone (based on number of fish present on this site compared to the total number of farmed fish in that zone during this month/year).

Field	Description
dfozone	One of seven fish health zone codes
year	The year to which the median load data relate (yyyy)
month	The month for which the median load data have been estimated (mm)
chal_load_median^*^	Median monthly load of sea lice in the chalimus stages
lep_mot_load_median^*^	Median monthly load of *L. salmonis* in the motile stage (including AF)
lep_af_load_median^*^	Median monthly load of *L. salmonis* in the adult female stage
cal_mot_load_median^*^	Median monthly load of *Caligus* sea lice in the motile stage

^*^for each of these sea lice stages, the associated mean abundance value for the given year/month is multiplied by the number of fish present on a given farm to estimate the farm ‘load’. The median value is then taken from all the farms reporting such ‘load’ estimates in each DFO zone for a given year/month and reported in this dataset.

The summary statistics associated with key continuous variables from these various datasets are presented in Table 5. The dataset is provided as five separate files in CSV format and is published at the following *figshare* data repository (10.6084/m9.figshare.28078100)^26^. The code to process these data sets can be found at – https://github.com/modailmara/BCSalmonData.

**Table 5:** Descriptive summaries of key continuous variables in these datasets (NA = missing value).

Field	Dataset	N	NA	Min	Max	Mean	Median
length (mm)	all_wild_fish_lice	368,150	2.3%	3	553	53.3	47.0
weight (g)	all_wild_fish_lice	229,000	39.2%	0.1	1100	3.0	0.96
height (mm)	all_wild_fish_lice	127,844	66.1%	1	39	8.5	7.9
Any_louse *	all_wild_fish_lice	376,764	—	0	384	0.90	0
chalimus_ab	industry_farm_abundance	10,159	1.6%**	0	46.4	0.96	0.17
lep_motile_ab	industry_farm_abundance	10,159	1.6%**	0	53.4	1.63	0.57
lep_af_ab	industry_farm_abundance	10,159	1.6%**	0	27.8	0.77	0.21
cal_motile_ab	industry_farm_abundance	10,159	1.6%**	0	41.5	0.41	0.06

^*^All twelve fields relating to the various sea lice species and stages were summed into a single value, to provide a total for ***any*** louse that was observed on a wild fish. This distribution is highly skewed; indeed only 28% of all the wild fish observed were found to have *any* sea louse infestation, with around 1% of the fish observed having over 10 sea lice.

^**^All 10,159 rows include farm abundance estimates for the various sea lice stages. However, for 162 entries the ‘weight’ feature could not be estimated due to missing farm inventory data. As such these rows (1.6% of total) cannot be included in any calculations used to estimate *weighted* values.

## Technical Validation

To explore the validity of the data, a summary of the physical characteristics of the wild fish sampled across the various zones and through the season is presented in Table 6. There is a clear progression in size over the sampled months with a gradual increase from March to May, before a more rapid increase in June (which extended into July, though the sample sizes are much more limited from this final month so mean values are not shown). The mean weight values recorded are in line with those shown in Jones and Hargreaves^27^, though the time periods reported in that paper cross monthly boundaries. It can also be seen that, as might be expected, when both Pacific salmon species were sampled concurrently, the chum tended on average to be a little longer/heavier than the pink salmon; again, the limited sample sizes later in the season make this less obvious in some zones.

**Table 6:** Breakdown of numbers of wild chum and pink salmon by DFO zone, with a summary of their mean sizes over the months March to June.

DFO Zone	Number of fish sampled				Mean length of fish (mm)				Mean weight of fish (g)			
	March	April	May	June	Mar	Apr	May	June	Mar	Apr	May	June
Chum	14,561	66,031	78,691	24,667								
2.3	2,394	12,445	8,918	504	37.8	41.4	48.0	56.1	0.5	0.7	1.1	2.1
2.4	7,592	15,066	10,258	736	38.4	39.9	44.0	55.7	0.5	0.7	1.0	2.4
3.1	581	2,546	1,396	—	35.2	37.4	40.5	—	0.5	0.7	0.9	—
3.2	—	2,477	2,951	1,455	—	38.6	56.5	104	—	0.6	2.8	11.8
3.3	3,992	31,035	48,906	19,803	37.5	42.3	53.3	75.8	0.5	0.8	2.5	7.2
3.4	2	600	538	129	—	44.4	54.2	55.9	—	1.0	2.7	2.3
3.5	—	1,862	5,724	2,040	—	41.3	45.1	55.5	—	0.8	1.1	2.2
Pink*	**4,785**	**41,814**	**65,309**	**35,043**								
3.1	144	1,222	720	—	31.6	36.4	40.6	—	0.4	0.6	0.9	—
3.2	—	2,029	2,004	1,087	—	33.9	52.7	86.1	—	0.4	2.1	6.8
3.3	4,630	34,044	49,986	28,198	32.2	37.7	51.4	71.8	0.3	0.5	1.9	4.8
3.4	11	2,307	2,019	403	31.3	35.8	48.8	64.7	0.3	0.5	1.4	3.7
3.5	—	2,212	10,580	5,355	—	38.4	44.3	56.9	—	0.6	1.2	2.6

^*^In Zones 2.3 and 2.4, over all years, only a total of 18 pink salmon were recorded, as such these are not reported in this table.

Note that just over 60% of samples recorded the weight of the fish. However, for some measures of risk, sea lice density (measured in lice/g) is required, so it may be useful to estimate the weight for those fish that have only length data. The association between length and weight is subject to a degree of variability, but it has been demonstrated^21^ that it is possible to build relationships that provide reasonable estimates of fish weight for samples where length is present.

## Usage Notes

With data from over 350,000 individual wild salmonid hosts and sea lice counts from an estimated 2 M farmed fish, over a two decade period, it may be tempting to assume that all the questions associated with sea lice interactions between farmed and wild populations can be answered using these data. This would be a mistake. As has been noted, the spatial coverage across the BC coast is fairly sporadic as far as wild sampling activities are concerned. With the exception of the Broughton Archipelago (Zone 3.3) and to a more limited extent the Central Coast area (Klemtu, Zone 3.5), other zones do not have extensive temporal coverage.

Even where data do exist, care must be taken to ensure that differences in, say, sampling protocol are adequately factored into any analyses that are carried out. It was noted that two very different sampling protocols have been used when estimating the sea louse infestation levels on wild fish in this dataset. As an illustration of the issues that may arise as a result, a summary of the estimated sea louse prevalence (proportion of fish with any sea louse infestation) and intensity (the mean number of sea lice on each infested fish) on wild chum and pink is shown in Table 7 for 2008 and 2009 on fish sampled under the ‘non-lethal’ and ‘lethal’ sampling protocols respectively. As the ‘non-lethal’ protocol was only used in Zone 3.3, the summarised data relate only to fish from that zone, with 2008 and 2009 being selected as there were relatively large sample sizes for all the months being compared.

**Table 7:** Estimates of sea louse prevalence and intensity on wild chum and pink in 2008 (N = 30,240) and 2009 (N = 26,371) for fish sampled under ‘non-lethal’ and ‘lethal’ sampling protocols respectively in sub-zone 3.3.

*Non-lethal*	2008			2009			
	Apr	May	June	Apr	May	June	July
All fish	5669	9451	7997	3174	9929	5495	528
Not infested	5216	8160	5978	3049	8160	4489	396
Infested	453	1291	2019	125	1769	1006	132
All lice	504	1738	3114	128	2322	1386	187
Prevalence	8.0%	13.7%	25.2%	3.9%	17.8%	18.3%	25.0%
Intensity	1.11	1.35	1.54	1.02	1.31	1.38	1.42
***Lethal***							
All fish	1432	3348	2343	1092	2988	2904	261
Not infested	1374	3138	2025	1070	2776	2486	206
Infested	58	210	318	22	212	418	55
All lice	70	302	494	26	251	776	104
Prevalence	4.1%	6.3%	13.6%	2.0%	7.1%	14.4%	21.1%
Intensity	1.21	1.44	1.55	1.18	1.18	1.86	1.89
**Compare Lethal / Non-lethal**							
Prevalence	51%	46%	54%	51%	40%	79%	84%
Intensity	109%	107%	101%	116%	90%	135%	133%

As might be expected there are differences in the estimates generated under these differing protocols. In addition to the fact that the method of sampling fish and counting sea lice differed, the sampling events do not entirely overlap in terms of either the exact time in each month at which they were taken, nor in terms of spatial extent. What is clear is that similar patterns can be seen under both protocols. For example, infestation levels tend to rise over the course of the season. It can also be seen, particularly in 2008, that the prevalence levels estimated using the lethal method were around half those estimated using the non-lethal approach. However, when considering intensity, the levels reported under the lethal approach were slightly higher. This pattern appears to be repeated in 2009 with the exception of May where the intensity estimate for the non-lethal sampling is slightly higher, though in this month the prevalence difference reported from the lethal samples is also substantially lower. Indeed, there appears to be a strong correlation between these two metrics, in that as the overall prevalence estimates in the lethal sampling rise so the proportional difference in levels of intensity also increase.

The data shown in Table 7, have aggregated all sea lice into a single number, without reference to the stage or species that might be involved. This is partly due to the fact that different levels of granularity of description exist across assessment protocols and that these have in some cases changed over the two decades. Using the simple “any louse” categorisation allows the analyses to disregard these subtleties when exploring initial trends. In the case of wild sampling, the vast majority of sea lice observed tend to be in the early (copepodid or chalimus) stages and as such the absence of detailed lice stage data within certain protocols is less problematic.

It is not the purpose of this paper to delve into the details of such limitations or to attempt to explain the mechanisms that might cause differences in their interpretation. It is rather to illustrate that common patterns do exist, but also to make it clear that reported sea lice infestation metrics will tend to differ according to the protocols being used. Much more detailed analyses are required to generate comprehensive explanatory narratives and one of the intentions in making this dataset available is to enable such explorations to be carried out.

## Supplementary information

Supplementary Information

## Data Availability

The Python code used to integrate the disparate data sets as well as to prepare the graphs and tables included in this paper can be found at – https://github.com/modailmara/BCSalmonData.
