# Correction: Sea lice infestation dataset for wild and farmed salmon populations on the Pacific coast of Canada (2001–2023)

**DOI:** 10.1038/s41597-026-06832-0

**Published:** 2026-02-11

**Authors:** Crawford W. Revie, Thitiwan Patanasatienkul, Gregor McEwan, Martin Krkošek, Lance Stewardson

**Affiliations:** 1 Department of Computer and Information Sciences, University of Strathclyde, 26 Richmond Street, Glasgow, G1 1XQ Scotland UK; 2 Department of Health Management, University of Prince Edward Island, 550 University Avenue, Charlottetown, Prince Edward Island C1A 4P3 Canada; 3 WOAH Regional Representation for Asia and the Pacific, Bunkyo-Ku, Tokyo Japan; 4 Modail Mara, Charlottetown, PEI Canada; 5 Department of Ecology and Evolutionary Biology, University of Toronto, 1310 Marwalk Cres, Toronto, ON M5S 3B2 Canada; 6 Mainstream Biological Consulting, 1310 Marwalk Cres, Campbell River, BC Canada

Correction to: *Scientific Data* 10.1038/s41597-025-05653-x, published online 31 July 2025.

In the version of this article initially published, Martin Krkošek (Department of Ecology and Evolutionary Biology, University of Toronto, Canada) was not included in the author list and now appears in the HTML and PDF versions of the article.

