# Supplementary material for: Sea lice infestation dataset for wild and farmed salmon populations on the Pacific coast of Canada (2001–2023)
Source: Sci Data. 2025 Jul 31;12:1331. doi: 10.1038/s41597-025-05653-x (PMC12313945; doi:10.1038/s41597-025-05653-x)
Supplement: Supplementary file 1 — Supplementary Information [file 41597_2025_5653_MOESM1_ESM.pdf]

Sea lice infestation dataset for wild and farmed salmon populations on the Pacific coast of Canada (2001-2023)

Crawford W. Revie, Thitiwan Patanasatienkul, Gregor McEwan, Lance Stewardson

Supplementary Information

To generate the dataset collected for this study, wild juvenile salmonid sampling events and sea lice counts were integrated from ten different programmes, each with slightly differing protocols. In **Table S1** each of these is noted in broad outline and, where available, relevant reference points from which further details can be obtained are provided.

Programme	Code	Type	Protocol
Salmon Coast Station	SCS	Non-lethal	This is the longest running of the 'non-lethal' sampling programmes. Over the past two decades it has broadly followed the same protocol. In each year the same 3 sites are targeted (Glacier, Burdwood, Wicklow within sub-zone 3.3) with a beach seine used to sample from the near-shore environment. The target in most years was to sample 50 each of chum and pink juveniles. This protocol is characterised as "non-lethal" due to the fact that from 2005 onwards the sea lice enumeration has been based on visual inspection of live fish captured in clear plastic envelopes made from Ziploc® bags using a 16x magnification hand lens. However, in the earliest years (2001 to 2004) the fish were frozen and these samples were analysed in the laboratory (i.e. similar to the "lethal" approaches noted below).<br><br>An excellent and in-depth description of the methods adopted within this protocol, including various changes in approaches and species identification, can be found at the following site: https://github.com/salmoncoast/Sea-lice-database
Martin Krkošek	MK	Non-lethal	These cover a set of sampling activities that followed a similar approach to that used by the 'SCS' protocol (above), but covering a wider geographical area, across much of the Broughton Archipelago (sub-zone 3.3), and was carried out between 2003 and 2009 under the leadership of Dr. Martin Krkošek. This was a more research-oriented set of activities. This included analysis to explore the robustness of the 'non-lethal' approach, which was subsequently adopted by other protocols. Details at: https://doi.org/10.1577/T04-133.1
Hakai	Hak	Mixed	The data from the Hakai Institute comes from a larger research programme on, "the early life history of juvenile salmon in coastal British Columbia". This includes topics such as aquatic habitat, migration routes and timings; indeed "parasite infection dynamics" are only one of six major areas of focus. As such the collection and sea lice enumeration protocols varied, by study, over the years. In general, a non-lethal approach, similar to the 'SCS' model was adopted for

			field observation, with a sub-set of the samples being euthanized and sent to a laboratory for assessment. For this reason, we have characterised data in the ‘Hak’ dataset as “Mixed”. More details on that programme can be found at: http://dx.doi.org/10.21966/1.566666
Cedar Coast	CC	Non-lethal	The Cedar Coast Field Station have run a series of sea lice monitoring activities based on wild juvenile salmon caught in the Clayoquot Sound region of BC. This programme has released data from 2018 to 2021, using a protocol largely in line with that adopted by ‘SCS’/’MK’. Details can be found at: https://github.com/CedarCoastFieldStation/Sea-lice-database/
Kitasoo	Kit	Lethal	Kitasoo/Xai'xais First Nation have had an established programme to monitor sea lice infestations on out-migrating wild juvenile salmon in their traditional territory since 2004. Unfortunately, the data from that initial year (reported in https://doi.org/10.1016/j.fishres.2007.11.018) could not be located, but consistent sampling from 2005 onwards is include from around 15 sites. Typically, between 50 to 100 juvenile salmon were sampled with a beach seine, euthanized, and frozen for later sea lice evaluation in the laboratory. This evaluation was carried out at the BC Centre for Aquatic Health Sciences (CAHS) in Campbell River, BC. More details can be found at: https://doi.org/10.1016/j.aquaculture.2011.07.018
Dept of Fisheries and Oceans	DFO	Lethal	A team of DFO scientists were involved in sampling between 2003 and 2009. Like ‘MK’ they operated across the Broughton but in a broader set of locations and habitats. They used both beach and purse seines and typically collected no more than 30 samples of each species, which were frozen and sea lice later enumerated in a DFO laboratory. Details can be found at: https://www.researchgate.net/publication/256803822_Pink_Salmon_Action_Plan_Sea_Lice_on_Juvenile_Salmon_and_on_Some_Non-salmonid_Species_in_the_Broughton_Archipelago_in_2003
Broughton Archipelago Programme	BAMP	Lethal	During the period 2010 to 2012 a number of researchers who had been engaged in the study of sea lice infestation on wild salmonids in the previous decade came together to run a cross-organisational set of surveys. (Formally including, the salmon farming companies operating in the Broughton Archipelago, Fisheries and Oceans Canada, university researchers, and the Coastal Alliance for Aquaculture Reform.) The 160 sites that had been part of the ‘MK’ programme were reduced to just under 100 sites in the Broughton, and then latterly to around 80 sites. A similar approach to sampling fish was adopted, but samples were frozen and submitted to the lab for enumeration. There were also aspects of research to the BAMP programme, so that samples were send to both the CAHS and DFO laboratories for comparison, and various in-

			field comparisons were made to look at issues such as inter-rater consistency. Details on various aspects of BAMP can be found at: https://doi.org/10.3354/dao02616
Marine Environmental Research Program	MERP	Lethal	This was less of a specific 'protocol' than it is a reference to a BCSFA-sponsored project (under their MERP framework) to bring together existing sea lice monitoring data that existed outside of the Broughton prior to 2017. In most cases the studies included came from the west coast of Vancouver Island and adopted the 'lethal' approach. There were also some data from the Port Hardy area integrated into this project from 2011 to 2016.<br><br>Indeed, it is somewhat confusing the refer to this sampling, particularly after 2010 as "MERP". The Port Hardy (sub-zone 3.4) data were in fact collected by Pacificus Biological Services from 2011 (see also below), while much of the sampling on Vancouver Island involved Mainstream Biological Consulting (again see below). However, the MERP project was the first time that these data, together with the various programmes from the Broughton were brought together into a single dataset (and formed the basis for the expanded data that are reported in this paper).
Pacificus Biological Services	Pacif	Lethal	As mentioned above, sampling in the Port Hardy area (sub-zone 3.4) has been carried out since 2011 by the Pacificus group. The earlier sampling is reported under the "MERP" label – see above – as this was the project under which the data were initially integrated into the wider dataset. The sampling approach followed a similar 'lethal' approach to that discussed in the "BAMP" protocol. Details can be found at: https://mowi.com/caw/wp-content/uploads/sites/7/2022/08/MOWI-Sea-Lice-Study-Goletas-Channel-2022-FINAL-V2.pdf
Mainstream Biological Consulting	MBC	Lethal	The data collected under the industry-sponsored wild sampling programme by MBC are among the most extensive both in terms of time-frame and geographical extent. Again the 'lethal' protocol was used, and many of the fish reported under the "MERP" heading were collected by MBC. There are annual reports from 2014 for at least six areas in BC that can be found on the company web sites. The methods used over the past decade, and across areas, have remained largely consistent over time. A recent report (from 2022 relating to sub-zone 3.3) provides a summary of these methods, at: https://www.cermaq.ca/assets/Discovery-Islands-Juvenile-Salmonid-Monitoring-2022.pdf

Table S1 – Brief descriptions of the programmes from which various data have been integrated into the current dataset of sea lice infestation on wild salmonids along the BC coast over the period 2001 to 2023, with references to relevant sources of more detailed descriptions.

As noted in the main text, we have included a copy of sea lice counts on salmon farms maintained by DFO, with some slight modifications to allow for easy cross-referencing to the monthly sea lice count data also included in this dataset. The data were downloaded from the “Industry sea lice counts on salmon farms” web page of the Government of Canada Open Government site on 17 March 2025 (https://open.canada.ca/data/en/dataset/3cafbe89-c98b-4b44-88f1-594e8d28838d).

We formatted these count data to match those in the <industry_farm_abundances.csv> file. This involved aligning the Facility Reference Number (DFO) with the facility_id to link to site descriptions in the <industry_farm_details.csv> file. The sea lice stage/species columns were also re-named to allow for direct comparison and/or linkage, while a number of other fields required separate reporting, as shown in **Table S2**.

It should be noted that these data were only collected by DFO from 2011 and as such there are no equivalent data from the period 2004 to 2010, for which data are present as monthly counts in the ‘industry’ sea lice dataset. In addition, it should be noted that five sites present in the original DFO download have been excluded from the file provided here. Four of these sites (Barkley, Bickley Bay, Cleagh Creek and Cormorant) had very few counts (only between 6 to 16 in total) which came from a single year of production. The fifth site (Glacial Creek) had more count records (N = 139) but as this was mostly used as a brood-stock site, less than 20% of these recorded actual sea lice estimates. As such, from the initial download of 18,808 rows, the modified file consists of a total a 18,625 rows, where each row represents the average results from a sea lice counting event, typically occurring on a weekly basis, at each aquaculture site with salmon in production across the BC coast.

Field	Description
facility_id	ID that associates each record with the farm from which the data comes. These were generated to allow for cross-reference to the data collected from 2004 in the ‘industry’ data files.
year	The year to which the abundance data relate (yyyy)
month	The month for which the abundance data have been estimated (mm)
day	The day of the month on which the sampling took place, where this was present (dd). In cases where only the month was reported this field is left blank.
fish_selected	The number of fish are not reported in the DFO dataset, this field has simply been left for consistency (see also next row)
num_pens_sampled	The number of pens that were involved in the sampling event is reported. The DFO definition for “sampling as per Conditions of Licence” involved a minimum of 3 pens with 20 fish per pen. Where this condition is not met that information in captured in the “comments” field. In cases where no such comment is present, it would be relatively safe to assume that the number of “fishselected” will be equal to (“number_of_pens” * 20); i.e. in the majority of cases, a total of 60 fish.
chalimus_ab	Mean abundance of sea lice in any stage prior to becoming motiles (sometimes referred to as “juvenile stages”)
lep_motile_ab	Mean abundance of *L. salmonis* in all motile stages (including *L. salmonis* in the adult female stage)

lep_af_ab	Mean abundance of *L. salmonis* in the adult female stage
cal_motile_ab	Mean abundance of *Caligus* sea lice in the motile stage. The DFO description notes *Caligus clemensi*, but in reality, no checking to confirm which sub-class these sea lice may belong to is carried out, so we chose both here and in the other datasets to simple note *Caligus*.
sample_type	The 'reason' associated with this particular sampling event. In the majority of cases (~75%) this is "routine monitoring", but could also be due to "follow up" from an exceedance report, be linked to various types of treatment events, etc. A full description of the possible contents of this, and other fields, and their correct interpretation can be found at the DFO web site noted (under "Terminology").
comments	This field contains narrative text that can be used to better understand the sampling events; for example, why no counts were reported ("fallow", "harvesting", etc.).

Table S2 – Description of data fields associated with the mean sea lice abundance values reported by farms to DFO. [***DFO_farm_abundance***]
